# Unraveling the Formation of Gelatin Nanospheres by Means of Desolvation

**DOI:** 10.1021/acs.nanolett.3c03459

**Published:** 2023-11-15

**Authors:** Negar Hassani Besheli, Martijn Martens, Elena Macías-Sánchez, Jos Olijve, Fang Yang, Nico Sommerdijk, Sander C. G. Leeuwenburgh

**Affiliations:** † Department of Dentistry-Regenerative Biomaterials, Radboud University Medical Center, 6525 EX Nijmegen, The Netherlands; ‡ Department of Medical BioSciences, Radboud University Medical Center, Geert-Grooteplein Zuid 28, 6525 GA Nijmegen, The Netherlands; § Electron Microscopy Centre Radboudumc, Technology Center Microscopy, Radboud University Medical Center, Geert-Grooteplein Noord 29, 6525 GA Nijmegen, The Netherlands; ∥ Department of Stratigraphy and Paleontology, University of Granada, Avenida de la Fuente Nueva S/N, CP 18071 Granada, Spain; ⊥ Rousselot BV, Port Arthurlaan 173, 9000 Ghent, Belgium

**Keywords:** gelatin nanoparticles, desolvation, self-assembly, cryo-TEM, nanoparticle formation

## Abstract

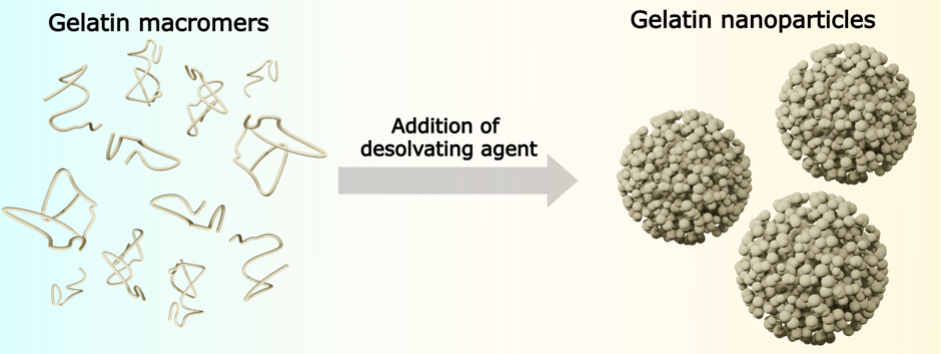

Gelatin nanoparticles (GNPs) have been widely studied for a plethora of biomedical applications, but their formation mechanism remains poorly understood, which precludes precise control over their physicochemical properties. This leads to time-consuming parameter adjustments without a fundamental grasp of the underlying mechanism. Here, we analyze and visualize in a time-resolved manner the mechanism by which GNPs are formed during desolvation of gelatin as a function of gelatin molecular weight and type of desolvating agent. Through various analytical and imaging techniques, we unveil a multistage process that is initiated by the formation of primary particles that are ∼18 nm in diameter (wet state). These primary particles subsequently assemble into colloidally stable GNPs with a raspberry-like structure and a hydrodynamic diameter of ∼300 nm. Our results create a basic understanding of the formation mechanism of gelatin nanoparticles, which opens new opportunities for precisely tuning their physicochemical and biofunctional properties.

During recent decades, protein-based nanoparticles have attracted considerable research interest due to their broad applicability in, e.g., drug delivery, vaccine development, and biocatalysis. Gelatin nanoparticles (GNPs) are particularly attractive protein-based nanoparticles for biomedical applications because gelatin is polyampholytic, water-soluble, biodegradable, biocompatible, cell-stimulatory, thermoreversible gel formation, poorly antigenic, abundantly available from various sources, and cost-effective. Additionally, gelatin offers versatile functionalization opportunities due to the presence of several functional groups for loading various pharmaceutical excipients and targeting ligands.^1−5^

Several techniques have been implemented to produce GNPs, including emulsification, solvent evaporation, reverse-phase microemulsion, nanoprecipitation, a self-assembly method employing an infrared lamp, and desolvation. The latter desolvation method has meanwhile emerged as the most commonly employed method for synthesizing GNPs due to its low cost and ease of particle synthesis.^3,6,7^ A fundamental understanding of the self-assembly of gelatin macromers into GNPs during desolvation is crucial to achieving precise control over particle synthesis. Such control would facilitate careful tuning of the physicochemical properties of GNPs, which in turn determine their biological functionality in terms of colloidal stability, drug loading capacity, drug release kinetics, cell internalization capacity, and biodistribution profile.^8^ Controlling the physicochemical properties (e.g., size) of GNPs prepared using desolvation also allows one to tailor the mechanical properties of colloidal gels composed of these GNPs. These colloidal gelatin gels show enhanced rheological performance in terms of accelerated stress relaxation at high strains as well as superior shear-thinning and self-healing behavior as compared to that of traditional monolithic hydrogels.^9−11^ These attractive features create new opportunities for applications in tissue engineering and regenerative medicine.

Several studies have already reported that both compositional (type of desolvating agent, gelatin concentration, etc.) and processing parameters (temperature, pH, etc.) of the desolvation process determine the final properties of GNPs in terms of, e.g., their size and dispersity.^12−17^ Generally, proteins tend to sequester their hydrophobic residues in their core and expose their polar residues to the aqueous environment under normal physiological conditions. This behavior is crucial for proper protein folding and enhances protein solubility in aqueous environments. However, the addition of poor solvents such as acetone disturbs the balance between electrostatic and hydrophobic interactions, thereby inducing phase separation and protein precipitation.^13,18^

Joye et al. provided a general description of the various stages of the desolvation process, including the creation of supersaturation, leading to particle nucleation and growth. Upon adjustment of the ratio of a desolvating agent to the solvent, the protein concentration of the resulting system can exceed the equilibrium saturation concentration (*C*_eq_) to create supersaturation, inducing the formation of small nuclei due to dynamic and stochastic association of macromolecules. Subsequently, nuclei can grow by either condensation or coagulation, resulting in larger structures that ultimately form the final protein nanoparticles.^19^ Generally, GNPs synthesized by desolvation are widely employed for numerous biomedical applications.^5,12,20,21^ However, despite several decades of research on the synthesis and characterization of GNPs, the underlying mechanism by which gelatin macromers self-assemble into nanoparticles upon desolvation remains poorly understood and validated. This lack of a fundamental understanding can be mainly attributed to the fact that time-resolved physicochemical analysis and visualization of nanoscale gelatin particles in aqueous and organic solvent mixtures are highly challenging, which hinders visual in situ monitoring of the formation of GNPS during desolvation. Such time-resolved in situ monitoring of GNPs during their formation by desolvation is urgently required to fundamentally understand the formation mechanism.

Here, we combine complementary analytical and advanced imaging techniques, including dynamic light scattering (DLS), turbidimetry, scanning electron microscopy (SEM), and cryogenic transmission electron microscopy (cryo-TEM), to monitor, for the first time, the process of GNP formation in a time-resolved manner and unravel the underlying nanoparticle formation mechanism during desolvation. Our data demonstrate that the formation of GNPs starts with the formation of primary particles upon gradual addition of the desolvating agent, which results in reduced charge screening upon removal of the water surrounding gelatin macromers. The formation of these primary particles is mainly driven by enhanced attractive electrostatic molecular interactions between oppositely charged gelatin residues. Subsequently, these primary particles self-assemble by coagulation into colloidally stable GNPs with a raspberry-like morphology and a diameter of ∼300 nm. The obtained results provide a fundamental understanding of GNP formation during desolvation, opening new opportunities to precisely tune their physicochemical and biofunctional properties.

GNPs were synthesized by desolvation of an aqueous gelatin solution through gradual addition of acetone followed by glutaraldehyde cross-linking, which resulted in a cross-linking density of 35.6 ± 1.9%. SEM and cryo-TEM imaging confirmed the spherical morphology of GNPs in dehydrated and hydrated states, respectively (Figure 1a,b). Importantly, these nanoparticles were observed to be self-assembled from smaller globular building blocks. GNPs had a negative surface charge of −13.9 ± 0.4 mV (Table S3) and an average diameter of 200 ± 84 nm in dry state (Figure 1c). In swollen state, on the other hand, GNPs had a considerably larger hydrodynamic diameter of 340 ± 54 nm, as determined by DLS (Figure 1d) due to the highly hydrophilic nature of gelatin.^13^

**Figure 1:**
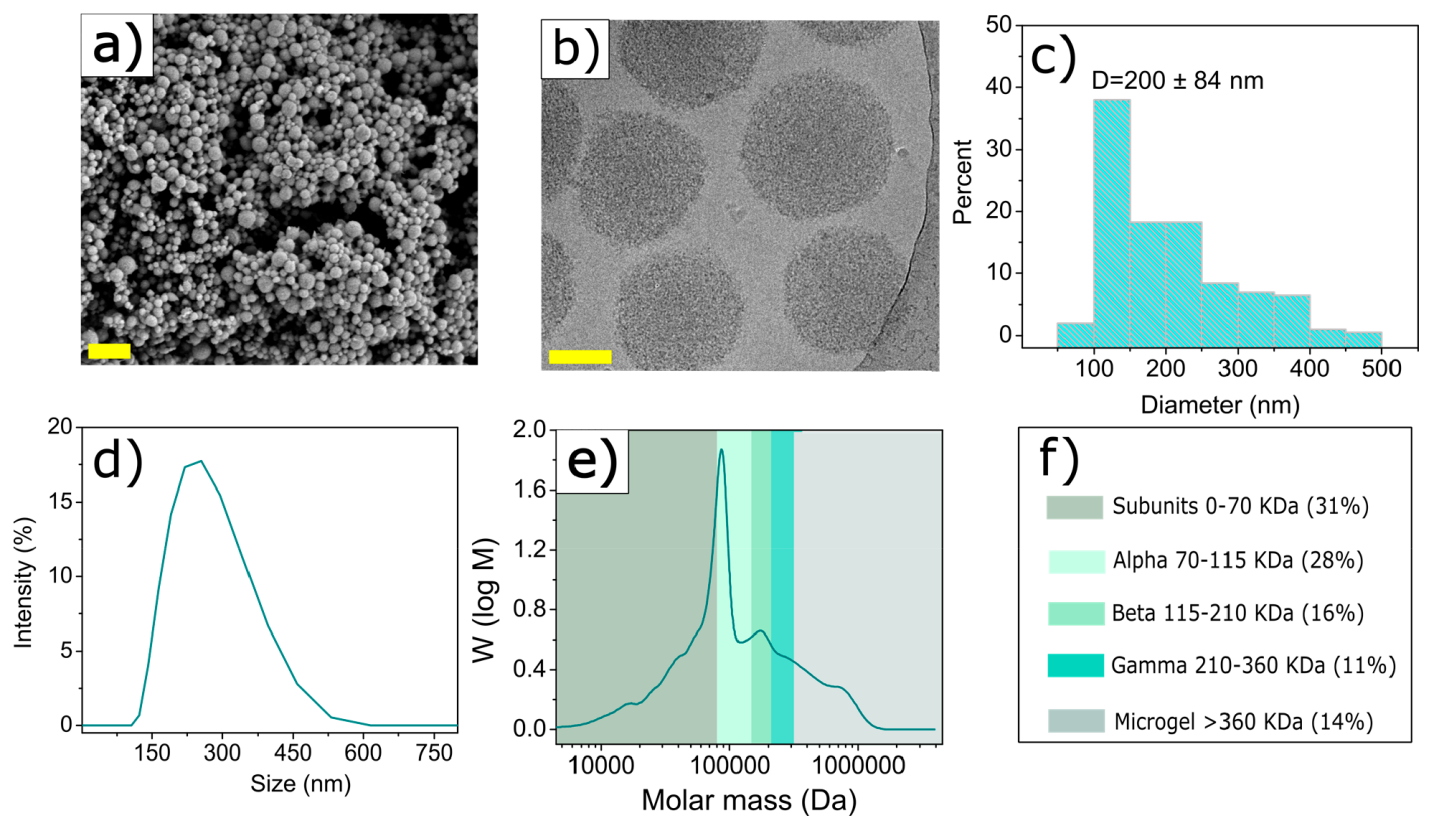
Gelatin nanoparticle characterization. (a) SEM image of lyophilized GNPs (the scale bar corresponds to 1 μm), (b) cryo-TEM image of water-swollen GNPs (the scale bar corresponds to 100 nm), (c) size distribution of dry GNPs obtained from SEM imaging, (d) size distribution of water-swollen GNPs obtained by DLS, (e) molecular weight distribution of standard gelatin (*M*_w_ = 179 kDa, type B), and (f) corresponding weight fractions of standard gelatin macromers used for GNP synthesis.

The heterogeneous particle size distribution observed by SEM and cryo-TEM imaging corresponds to the heterogeneous molecular weight distribution of gelatin macromers.^15^ GPC analysis indicated a broad and heterogeneous molecular weight distribution [ranging from 0 to 2000 kDa (Figure 1e,f)] of gelatin used and with a weight-average molecular weight (*M*_w_) of 179 kDa. Figure 1f shows the weight fractions of the gelatin macromers,^22^ revealing large amounts of low-molecular weight gelatin macromers (∼31% subunit fractions) in tested gelatin, which mainly contributed to the polydispersity of the synthesized GNPs. These results are in line with a previous study confirming the formation of polydisperse nanoparticles at gelatin subunit contents of >20%.^23^

To deepen our understanding of the influence of molecular weight on the size and polydispersity of GNPs, we also prepared GNPs using gelatins with higher *M*_w_ values (229, 244, and 276 kDa) and smaller subunit fractions (Table S4). These gelatins formed monodisperse (PDI < 0.1) and smaller GNPs with a narrower size distribution when compared to our standard gelatin with a *M*_w_ of 179 kDa (Figure S1). We ultimately selected gelatins with the lowest [*M*_w_ = 179 kDa (LMW)] and highest [*M*_w_ = 276 kDa (HMW)] molecular weights for further analysis of the kinetics of gelatin nanoparticle formation.

To monitor the kinetics of gelatin nanoparticle formation, we quantified the turbidity of gelatin solutions in a time-resolved manner as a function of the volume of the desolvating agent. Before the addition of acetone (standard desolvating agent), two absorption peaks were detected at 230 and 280 nm, corresponding to π → π* transitions in the peptide bonds and aromatic side chains, respectively (Figure 2a).^24,25^ Addition of acetone gradually enhanced the turbidity of the gelatin solution caused by the formation of light-scattering nanoparticles (Figure 2a, inset).^13^ By plotting turbidity (at a fixed wavelength of 600 nm) versus the acetone volume fraction (ϕ) in the water/acetone mixture, we could clearly discern three distinct phases in the nanoparticle formation process (Figure 2b).

**Figure 2:**
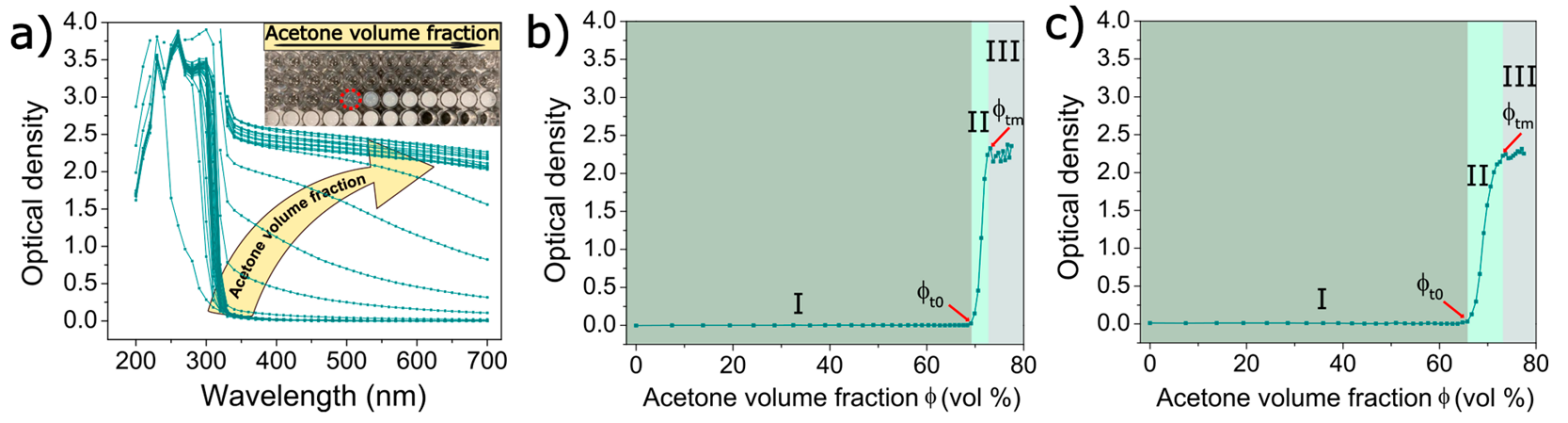
Turbidimetric studies of gelatin nanoparticle formation. (a) Ultraviolet–visible spectra of an aqueous gelatin solution (*M*_w_ = 179 kDa; type B) as a function of acetone addition (the inset shows turbidity of gelatin solutions upon gradual addition of acetone; the red dotted circle corresponds to the initial turbidity change). (b) Turbidity of the gelatin solution (*M*_w_ = 179 kDa; type B) at a fixed wavelength of 600 nm as a function of acetone volume fraction. (c) Turbidity of the gelatin solution (*M*_w_ = 276 kDa; type A) at a fixed wavelength of 600 nm as a function of acetone volume fraction. Acetone volume fractions corresponding to the onset (end of phase I) and end of the rapid turbidity increase (end of phase II) are denoted ϕ_t0_ and ϕ_tm_, respectively.

During the initial phase, denoted as phase I, gradual addition of acetone [ϕ = 0–67.5% (v/v)] did not affect the turbidity of the mixture, suggesting that light-scattering particles were still absent during this initial phase. As the acetone volume fraction reached approximately ϕ_t0_ = 68.3%, we observed a sudden 10-fold increase in the optical density of the gelatin solution, followed by a continuous increase in optical density at approximately ϕ_tm_ = 73.7% (where ϕ_tm_ refers to the acetone volume fraction corresponding to maximum turbidity). These changes were attributed to the growth of light-scattering GNPs (phase II).^26^ At a higher ϕ (>ϕ_tm_), the turbidity did not increase further, plateauing at a stable value. This plateau characterized phase III and indicates that further addition of acetone did not increase the number density of light-scattering particles. To deepen our understanding of gelatin nanoparticle formation, we also monitored the turbidity of gelatin solution of the highest *M*_w_ (276 kDa) as a function of ϕ. For this HMW gelatin, less acetone was required to induce nanoparticle formation, as evidenced by a shorter phase I in Figure 2c. On the contrary, more acetone was required to complete the growth phase because phase II was considerably broader than that of LMW gelatin. Additionally, growth rates of nanoparticles produced from HMW were lower (0.35) than those of LMW GNPs (0.67) (Table S5).

The kinetics of GNP formation was further studied by imaging the suspensions [photography (Figure 3a, inset)] and freeze-dried nanoparticles (SEM) (Figure 3a) as a function of acetone addition. At ϕ = 67.5% (v/v) (end of phase I), even though the acetone/water mixture remained transparent, there were already indications of aggregated spherical particles with an average dry size of ∼48 ± 17 nm (Figure 3b). At a slightly higher acetone content of 68.3% (v/v) (phase II), suspensions were still largely transparent, but the freeze-dried spherical particles were larger and revealed a broader size distribution of 111 ± 37 nm. With increasing acetone contents, these primary particles acted as building blocks for the assembly of colloidally stable GNPs with sizes ranging from 218 ± 62 nm at 72% acetone to 303 ± 74 nm at 73.7% acetone. At the highest acetone content of 75.2% (phase III), particles reached sizes of ∼427 ± 118 nm, while the size distributions broadened considerably. These suspensions were colloidally stable without any sedimentation of the large aggregates. However, once the glutaraldehyde cross-linking reaction had reached completion, strong particle aggregation and resulting sedimentation of large gelatin deposits were observed (dotted red circles in Figure 3a), which were most likely caused by a combination of intra- and interparticle cross-linking. High-resolution SEM imaging of nanoparticles (on the order of tens of nanometers) produced by adding 72% acetone revealed that colloidally stable GNPs with raspberry-like morphologies (Figure 3c) were formed by self-assembly of 32 ± 5 nm primary particles as building blocks (Figure 3d). This size roughly corresponded to the size range of primary particles observed in phase I, although it should be noted that heavy agglomeration complicated the accurate size determination of these primary particles formed at 67.5% (Figure 3a), which may have caused the slight difference in primary particle size, as observed in Figure 3a versus Figure 3d. Similar morphological changes were observed for GNP made from HMW gelatin (Figure S2). However, smaller primary particles (25 ± 4 nm) formed in phase I, which led to the formation of smaller GNPs with a size of 140 ± 36 nm, displaying similar raspberry-like morphologies.

**Figure 3:**
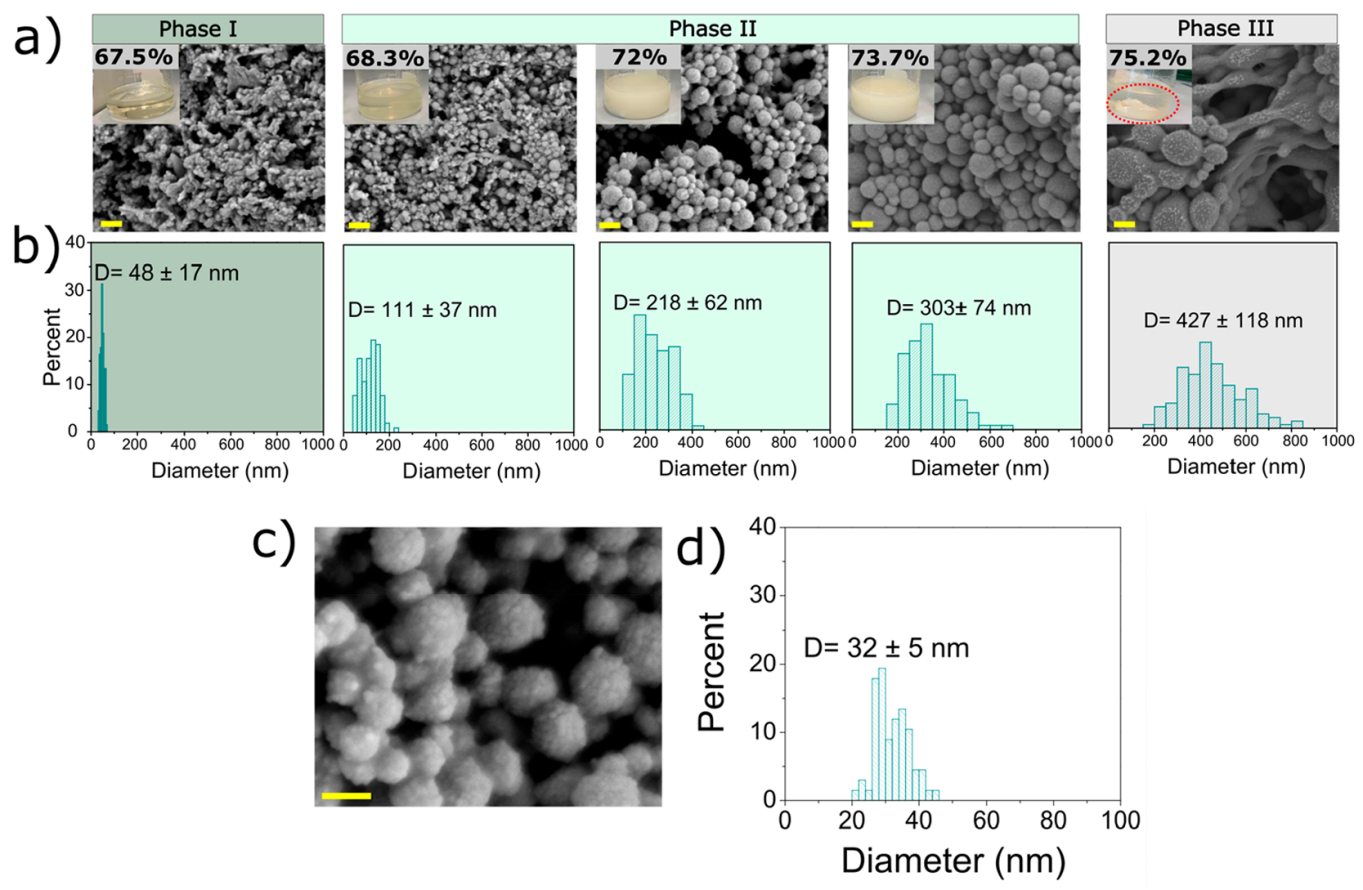
Imaging of gelatin nanoparticle formation. (a) Scanning electron micrographs of freeze-dried gelatin nanoparticles as a function of acetone content (the scale bars represent 400 nm) and photographic insets of gelatin solutions containing different amounts of acetone (the red dotted circle indicates gelatin sedimentation after the completion of cross-linking). (b) Size distribution of freeze-dried gelatin nanoparticles calculated from SEM images (shown in colors corresponding to the kinetic phases as identified using turbidimetric analysis). (c) High-resolution imaging of GNPs formed by the addition of 72% acetone showing raspberry-like structures (the scale bar represents 200 nm). (d) Size distribution of primary building blocks of GNPs.

High-resolution SEM imaging provided valuable information about the mechanism of GNP formation. However, dehydration during sample preparation most likely affects the dimensions and morphology of the original samples.^27^ Therefore, cryo-TEM was employed to visualize the GNP formation process, for the first time, in their native hydrated and un-cross-linked state. We visualized GNPs at three crucial stages of particle formation, i.e., (i) initial acetone-free aqueous gelatin solutions (start of phase I), (ii) gelatin solutions containing 67.5% acetone (end of phase I), and (iii) gelatin solutions containing 72% acetone (phase II). As shown in Figure 4a, no structural features were observed in a gelatin-free control sample consisting of an acetone/water mixture (67.5%). However, an aqueous gelatin solution [5% (w/w)] displayed a high number density of small features of <10 nm (Figure 4b) corresponding to dissolved gelatin macromers, which was in agreement with our DLS measurements of aqueous gelatin solutions showing peaks at ∼20 nm (Figure S3). In line with our SEM observations, cryo-TEM imaging confirmed the formation of small amounts of ∼18 ± 3 nm primary particles in a wet state upon the addition of 67.5% (v/v) acetone (Figure 4c, red arrowheads). With an increase in acetone content to 72%, these primary particles aggregated into spherically shaped particle clusters with a final size of 306 ± 51 nm as precursors for the final GNPs with raspberry-like morphology. These data provide the first visual evidence that GNPs are formed by coagulation rather than condensation (Figure 4d).

**Figure 4:**
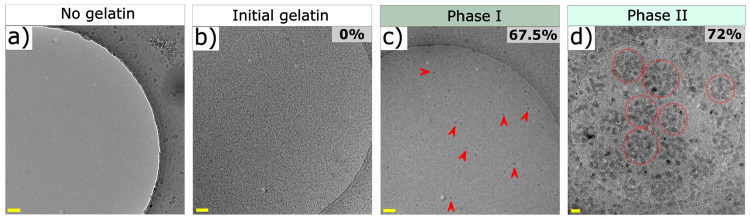
Visualization of the formation of gelatin nanoparticles in their native hydrated state using cryo-TEM. (a) Gelatin-free acetone/water [67.5% (v/v) acetone] mixture (control). (b) Gelatin dissolved in water [5% (w/w)] prior to acetone addition. (c) Formation of small amounts of primary particles (indicated with red arrowheads) upon addition of 67.5% (v/v) acetone to an aqueous solution of gelatin [5% (w/w)]. (d) Final gelatin nanoparticles formed by aggregation of primary particles into spherically shaped particle clusters upon addition of 72% acetone. Dotted circles around these spherical particle clusters are added to guide the eye. Scale bars represent 100 nm.

In summary, our study reveals, for the first time, turbidimetric and visual evidence of the mechanism of GNP formation during gelatin desolvation. Gelatin, a polyampholyte polymer with a 1:1 ratio of positively and negatively charged residues,^28^ was dissolved in water and acidified to protonate the amines along the gelatin backbone. These positively charged moieties induce repulsive forces between gelatin macromers, preventing uncontrolled aggregation upon addition of a desolvating agent.^29^ The desolvating agent gradually expelled water surrounding gelatin macromers, leading to local supersaturation as the primary factor controlling GNP formation. Local supersaturation increased with gelatin molecular weight, which resulted in smaller sizes and growth rates for GNPs synthesized from HMW gelatins. This local supersaturation induced by a desolvating agent reduced the spatial expansion of gelatin macromers and led to their controlled collapse as nanoparticles.^19^ This collapse can also be attributed to a gradual reduction in electrostatic repulsive interactions between similarly charged residues along gelatin macromers. The dielectric constant (ε) of the aqueous/organic solvent mixtures as calculated from the Silberstein equation (Figure S4) showed a continuous decrease with an increase in the content of the desolvating agent, which diminished the charge screening effect of the water and facilitated stronger electrostatic attraction between oppositely charged residues. According to the Debye–Hückel theory, electrostatic interactions between oppositely charged particles are proportional to ε^–3/2^.^30−32^ Therefore, the formation of primary particles at the end of phase I can be attributed to a local neutralization process caused by the addition of a desolvating agent. Due to the small size of the primary particles (Figures 3a and 4c) relative to the wavelength of the incident light, no visual changes were observed regarding the optical density of the gelatin solution, as confirmed by our turbidimetric studies (Figure 2b, phase I). The addition of more acetone (ϕ = 68.3–73.7%) to the gelatin solution led to the formation of the raspberry-like structure of GNPs, which results from the self-assembly of small primary particles, which was observed under both dehydrated and hydrated conditions along with a continuous increase in gelatin solution turbidity in phase II (as shown in Figures 3c, 4d, and 2b). This observation confirms that the growth phase in GNPs is primarily driven by coagulation, instead of condensation, as a result of collisions caused by either Brownian motion, external force fields (vigorous mixing), or particle–particle interaction^19^ (Scheme 1). The collision frequency of primary particles may decrease in a HMW gelatin solution due to its higher viscosity compared to that of LMW gelatin, which can contribute to the slower growth rates as reported in Table S5.^33^

**Scheme 1:**
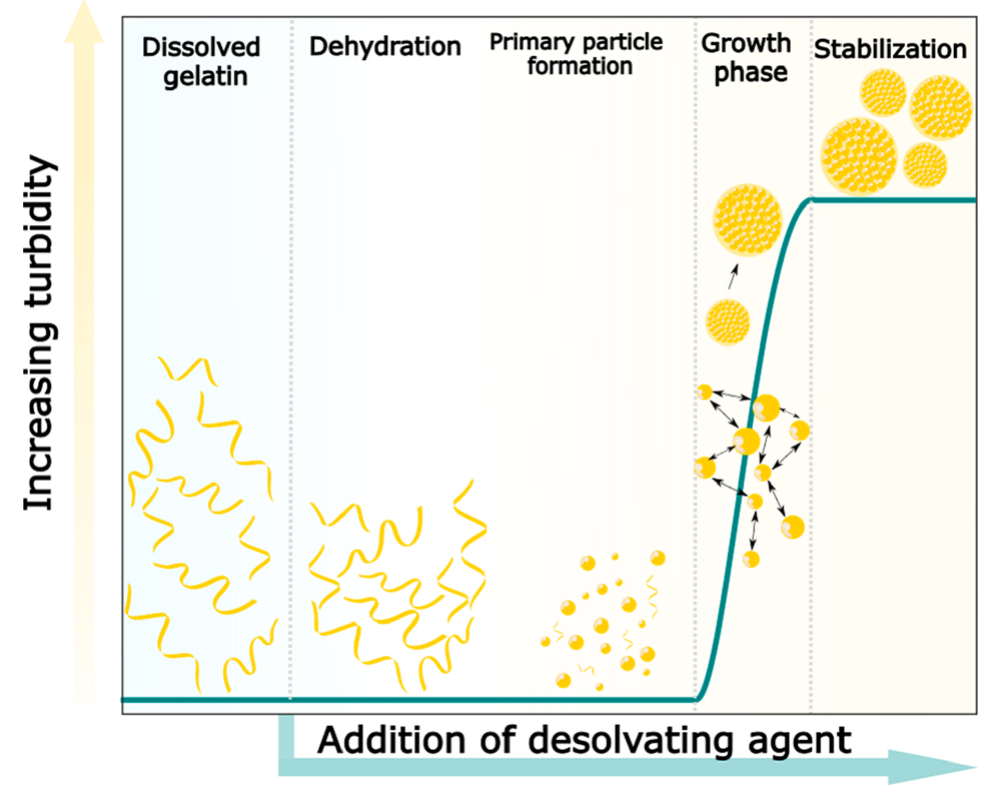
Schematic Representation of the Proposed Mechanism of Formation of GNPs by Means of Desolvation

We highlighted the crucial role of the molecular weight distribution of gelatin in controlling particle size and homogeneity. HMW gelatin macromers necessitate less desolvating agent to initiate GNP formation. Nucleation processes, such as GNP formation, are strongly influenced by the energy barrier required for the formation of nuclei, which is inversely proportional to supersaturation. HMW gelatin macromers typically create more supersaturation, reducing the energy barrier, accelerating nucleation, and resulting in smaller nanoparticles, as confirmed by Figure S2.^19^ Moreover, the higher chain flexibility of HMW gelatin macromers enhances macromer entanglement, leading to more compact particulate assemblies compared to LMW gelatin macromers.^34^ Consequently, the heterogeneous molecular weight of the gelatin chains leads to the formation of heterogeneous GNPs, as shown in Figure 1a–c.

The GNP formation mechanism, driven by local supersaturation and self-neutralization of charged residues, holds promise for predicting and precisely adjusting GNP physicochemical properties, offering controlled biofunctionality modulation. We validated this by replacing acetone with ethanol, which has a higher dielectric constant. Like that of acetone, gradual addition of ethanol altered the turbidity of the gelatin solution, dependent on the ethanol content (Figure 5a and inset). However, significantly larger amounts of ethanol (2.4-fold) were required to induce the formation of light-scattering particles in the mixtures (Figure 5b,c). This difference can be attributed to the higher dielectric constant of ethanol (ε = 24.3) and its greater polarity and protic nature compared to acetone (ε = 20.7).^35,36^ The increased dielectric constant of ethanol, while reducing the dielectric constant of the medium less than that of acetone at a fixed desolvating content (Figure S4), caused delayed dehydration of gelatin macromers. Notably, a larger ethanol volume fraction (84.8%) was necessary to initiate turbidity change at the end of phase I compared to acetone (68.3%), resulting in a lower dielectric constant of the resulting gelatin solution (32.5) compared to acetone addition (38.9). This lower dielectric constant promoted electrostatic attraction between oppositely charged residues (following the Debye–Hückel theory), leading to more compact assemblies upon local neutralization and the subsequent formation of smaller nanoparticles.

**Figure 5:**
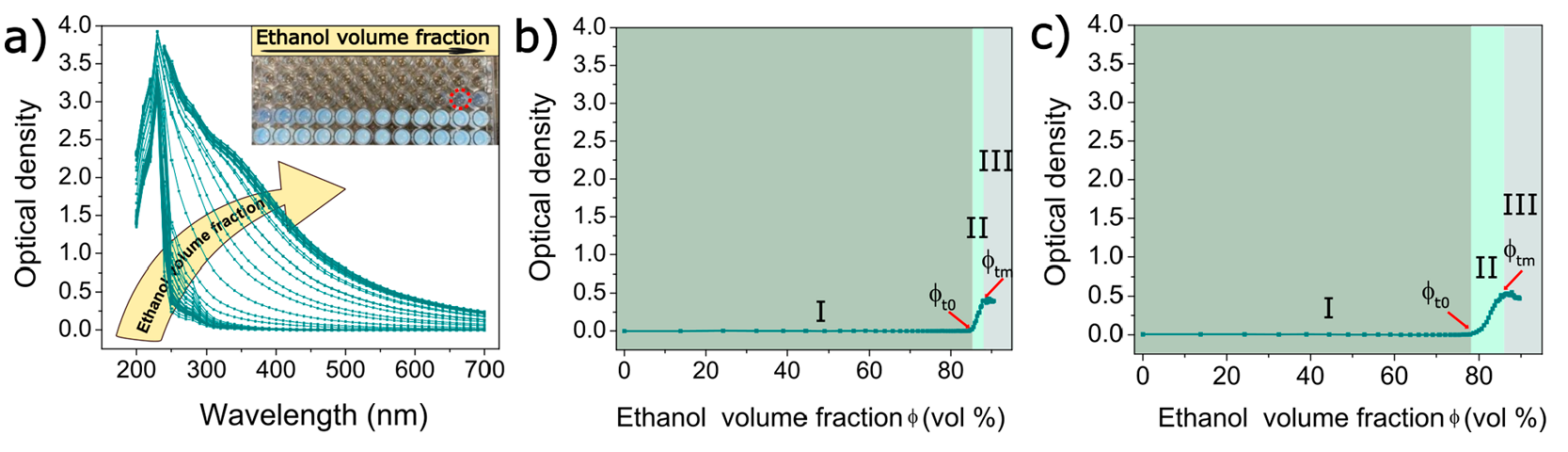
Turbidimetric studies of gelatin nanoparticle formation. (a) Ultraviolet–visible spectra of an aqueous gelatin solution (*M*_w_ = 179 kDa; type B) as a function of ethanol addition (the inset shows the turbidity of gelatin solutions upon gradual addition of ethanol; the red dotted circle corresponds to the initial turbidity change). (b) Turbidity of the gelatin solution (*M*_w_ = 179 kDa; type B) at a fixed wavelength of 600 nm as a function of ethanol volume fraction. (c) Turbidity of the gelatin solution (*M*_w_ = 276 kDa; type A) at a fixed wavelength of 600 nm as a function of ethanol volume fraction. Ethanol volume fractions corresponding to the onset (end of phase I) and end of the rapid turbidity increase (end of phase II) are denoted as ϕ_t0_ and ϕ_tm_, respectively.

Notably, our data provided support for this explanation as the addition of ethanol resulted in lower turbidity values compared to those with acetone, which points to the formation of smaller nanoparticles with reduced light-scattering activity,^26^ confirming the predictive power of the proposed mechanism. Figures S5 and S6 show that GNPs synthesized in ethanol/water mixtures were smaller than those from acetone. Similarly, GNPs from HMW gelatins required less ethanol, growing slower (0.08) than LMW gelatin-derived particles (0.13) (Figure 5c and Table S5). Consequently, GNP formation demanded more ethanol than acetone. A similar correlation between nanoparticle size and desolvating agent dielectric constant, noted in albumin desolvation, reaffirmed these findings.^37^ Overall, our data confirm that GNP formation relies on local supersaturation and self-neutralization of charged residues, mainly governed by the gelatin molecular weight and desolvating agent dielectric constant. The phase transitions observed during the formation of GNPs can serve as a valuable tool for tailoring the size of GNP to meet specific requirements regarding, e.g., colloidal stability, drug loading capacity, drug release kinetics, and cell internalization capability for applications in regenerative medicine and drug delivery.

High-resolution SEM and cryo-TEM imaging combined with turbidimetric analysis allowed us, for the first time, to demonstrate the different phases of gelatin nanoparticle formation by means of desolvation. The main driving force for GNP formation involves the creation of a locally supersaturated environment upon addition of the desolvating agent, which induces the self-neutralization of oppositely charged residues along gelatin macromers. These processes initiate the formation of primary particles, which act as building blocks of larger self-assembled GNPs. The primary particles further grow via coagulation to form raspberry-like nanoparticles with a size of ∼300 nm. This study provides a detailed overview of the mechanism of formation of GNPs in their native (wet) state. The basic insights obtained from this study will allow optimization of the synthesis of GNPs with tailored surface properties to maximize their biofunctional efficacy in biomedicine.

## Supporting Information Available

Detailed explanation of the synthesis and characterization procedures, GNP properties produced by the desolvation of two types of gelatins with different molecular weight distributions using acetone or ethanol, weight fractions of different gelatins and properties of the corresponding nanoparticles, a table comparing GNP formation kinetics between high- and low-molecular weight gelatin, SEM images of GNP formation at different volume fractions of acetone produced by HMW gelatin, size distribution of dissolved gelatin in water in the absence of acetone, and a figure depicting the dielectric constant of mixtures of acetone or ethanol with water as a function of acetone/ethanol volume fraction (PDF)
