# Supplementary material for: Unraveling the Formation of Gelatin Nanospheres by Means of Desolvation
Source: Nano Lett. 2023 Nov 15;23(23):11091–8. doi: 10.1021/acs.nanolett.3c03459 (PMC10722596; doi:10.1021/acs.nanolett.3c03459)
Supplement: Supplementary file 1 — nl3c03459_si_001.pdf [file nl3c03459_si_001.pdf]

Supporting information

Unraveling the formation of gelatin nanospheres by means of desolvation

Negar Hassani Besheli[1], Martijn Martens[2], Elena Macías Sánchez[3], Jos Olijve[4], Fang Yang[1], Nico Sommerdijk[2], Sander C.G. Leeuwenburgh*[1]

[1]Department of Dentistry-Regenerative Biomaterials, Radboud University Medical Center, 6525 EX Nijmegen, The Netherlands

[2]Department of Medical BioSciences, Radboud University Medical Center, Geert-Grooteplein Zuid 28, Nijmegen 6525 GA, The Netherlands; Electron Microscopy Centre Radboudumc, Technology Center Microscopy Radboud University Medical Center Geert-Grooteplein Noord 29, Nijmegen 6525 GA, The Netherlands

[3]Department of Medical BioSciences, Radboud University Medical Center, Geert-Grooteplein Zuid 28, Nijmegen 6525 GA, The Netherlands; Department of Stratigraphy and Paleontology University of Granada, Avenida de la Fuente Nueva S/N CP:18071 Granada, Granada Spain

[4]Rousselot BV, Port Arthurlaan 173, 9000 Gent, Belgium

1. Materials and methods

1.1. Gelatin nanoparticle synthesis

Gelatin with a weight-average molecular weight (Mw) of 179 kDa was kindly provided by Rousselot (Ghent, Belgium). This gelatin type was selected for standard synthesis of gelatin nanoparticles (GNPs) using the desolvation method, while acetone was selected as a standard desolvating agent. To initiate the desolvation process, 25 mL of a 5% w/w aqueous gelatin solution was prepared under shaking (500 rpm) at 50 ^{o}C. This initial gelatin solution concentration was selected as a commonly employed concentration for GNP production in desolvationg agent. Higher concentrations can lead to elevated viscosity and uncontrolled aggregation upon addition of acetone, while lower concentrations may promote the formation of smaller nanoparticles and lower particle yields [1]. The pH of the solution was then adjusted to 2.5 using 6M HCl (37% fuming, Merck). Subsequently, 64 mL of acetone was gradually added to the aqueous gelatin solution using a syringe pump at an injection rate of 4mL.min^{-1} under vigorous stirring (1000 rpm) at 40 ^{o}C. The resulting nanoparticles were stabilized by adding 158 µL of glutaraldehyde (25 wt/wt aqueous solution, Acros) at room temperature and allowing the crosslinking reaction to proceed overnight under stirring (500 rpm). The crosslinking reaction was then stopped by adding 100 mL of glycine solution (100 mM, Sigma-Aldrich). The resulting nanoparticles were collected by centrifugation (24000 rcf, 40 min) and

washed twice by redispersing them in demineralized water. Finally, the nanoparticles were dispersed in a mixture of acetone/water (1:3 v/v) and freeze-dried to obtain dry powder. To investigate the influence of gelatin molecular weight on the size and polydispersity of GNPs, GNPs were also synthesized from gelatins with higher Mw. For these HMW gelatins, a two-step desolvation process was applied to remove low molecular weight gelatin fractions more efficiently before the nanoparticle production. In the first desolvation step, 100 mL of acetone was rapidly added at once to 100 mL of gelatin solution (5% w/w) at a stirring speed of 1000 rpm. After the mixture was cooled down for 15 min, the supernatant was discarded, and the remaining high molecular weight gelatin was dissolved in 100 mL of demineralized water and freeze-dried for 48h. For GNP synthesis, a similar procedure was used as described above using the pretreated gelatin and a total volume of 58 mL of acetone as a desolvating agent. Table S 1 summarizes the specifications of the different gelatins that were tested.

Table S 1. Specification of gelatins utilized for the synthesis of GNPs.

Group	Origin of gelatin	Pretreatment method	Weight-average molecular weight (Mw, kDa)*
**Standard**	Bone	Acid-pretreated (type B)	179
**1**	Skin	Alkali-pretreated (type A)	229
**2**	Skin	Alkali-pretreated (type A)	244
**3**	Skin	Alkali-pretreated (type A)	276

* Mw of gelatin after the first desolvation step for gelatin type A.

1.2. Gelatin and gelatin nanoparticles characterization

The molecular weight distribution of gelatin was analyzed by High-performance size-exclusion chromatography (HP-SEC) analysis. First, an aqueous gelatin solution (2% w/v) was prepared at 60 ^{o}C. Then, 500 μL of this gelatin solution was mixed with 7.5 mL of eluent (1.0% w/v SDS with 0.1M Na_2SO_4 and 0.01M NaH_2PO_4 at pH 5.3), after which the resulting mixture was filtered through a 0.45 μm RC syringe filter. The HP-SEC was carried out at room temperature using a TSK G4000SWXL column, a Waters HPLC system including a Waters pump 2690 and a Waters 2487 UV detector.

The hydrodynamic size and polydispersity index (PDI) of nanoparticles were measured by Dynamic Light Scattering (DLS, Malvern Instrument Ltd) at 25°C, setting a minimum of 10 and a maximum of 100 runs per measurement. The measurements were carried out for well-dispersed aqueous particle dispersions in three replications (n=3). The zeta potential of synthesized nanoparticles was measured using a Zeta sizer (Malvern Instrument Ltd.). For this measurement, a well-dispersed particle dispersion a concentration of 1mg.mL^{-1} in HEPES (5mM, pH=7.4) was applied. Each experiment was conducted three times and data were presented as means ± standard deviations.

The morphology and size in dry state of all synthesized nanoparticles were studied using a Field Emission Scanning Electron Microscope (FE-SEM; Sigma 300, Zeiss, Germany). First, gelatin nanoparticles were freeze-dried in an acetone/water mixture (30/70 v/v%). Then, dried nanoparticles were placed on a double-stick carbon tape followed by coating with an electroconductive chromium layer with a thickness of ~ 10 nm. The average size and size distribution of nanoparticles were determined by measuring the diameter of at least 100 particles using ImageJ software.

The degree of crosslinking of gelatin nanoparticles was measured by a colorimetric 2,4,6-Trinitrobenzene Sulfonic Acid (TNBSA) assay [2]. Briefly, 5 mg of GNPs was dispersed in 1mL of $NaHCO_3$ solution (4% w/v, pH 8.5) and in 1mL of freshly prepared aqueous TNBS solution (0.5% w/v, Sigma-Aldrich). After 2h of incubation at 40°C, 2 mL of HCl (6M) was added to the reaction followed by 1.5 h incubation at 60°C. Subsequently, the sample was diluted with 4 mL of deionized water. Finally, 200 µL of the sample was transferred to a 96-well plate and the absorbance was measured at 346 nm using a UV-Vis spectrophotometer (Synergy HTX multi-mode reader, Biotek). The number of free primary amine groups present in gelatin nanoparticles after crosslinking relative to the primary amine groups before the crosslinking step was expressed as the degree of crosslinking.

1.3. Monitoring the kinetics of nanoparticle formation using UV-Vis spectrophotometry

To monitor the progress of GNP formation, nanoparticles were synthesized as described in section 4.1, and the optical density of gelatin solution (Mw=179 kDa) was measured upon addition of desolvating agent at various wavelengths between 200-700 nm using a UV-Vis-spectrophotometer. The synthesis was carried out by adding 100 mL of acetone at a rate of 4 $mL.min^{-1}$ to the aqueous gelatin solution, and every 30 seconds, 200 µL of the reaction mixture was transferred to a UVStar® 96 well-plate (Greiner, Bio one) to allow for recording the optical density. The obtained optical density at 600 nm was then plotted against the applied desolvating agent volume fraction (ϕ). The volume fraction of acetone at which the optical density of the gelatin solution suddenly increased 10-fold was defined as ϕ_{t0}, while ϕ_{tm} corresponded to the volume fraction of acetone above which the optical density of the gelatin solution increased less than 5%. The growth rate of GNPs was calculated by calculating the slope of linear fits between ϕ_{t0} and ϕ_{tm} in the obtained kinetic plots. To study the effect of higher Mw on GNP formation, gelatin with a Mw of 276 kDa was also employed to prepare GNPs, while ethanol was also used as alternative desolvating agent to study the effect of these antisolvents on the process of GNP. To this end, 240 mL of ethanol was added to the solutions of either HMw or LMw gelatins at an injection rate of 4 $mL.min^{-1}$. The optical density of the gelatin solution was measured every minute using a UV-Vis spectrophotometer. Finally, the effect of the addition of the specific desolvating agent on the dielectric constant of the resulting solvent mixture was calculated by means of the Silberstein equation [3].

$\varepsilon = \varepsilon_1 \nu_1 + \varepsilon_2 \nu_2$

where ε is the dielectric constant of the mixture, ε_1 and ε_2 refer to the dielectric constant of each component of the respective mixtures (i.e., 78.3, 20.7, and 24.3 for water, acetone, and ethanol, respectively), and ν_1 and ν_2 correspond to the volume fraction of each component.

1.4. Scanning electron microscopy

Scanning electron microscopy (SEM) was performed to visualize morphological changes of GNPs in dry state throughout the formation process. The synthesis was performed according to the method described in section 4.1. However, the synthesis as described in section 4.1 was stopped at five different acetone volume fractions which were selected from the study on GNP formation kinetics (section 4.3). The resulting samples were stabilized by glutaraldehyde followed by washing and freeze-drying prior to imaging. Image J software was used to measure the particle size and distribution in their dehydrated state by measuring the size of at least 100 particles. This experiment was performed using HMw gelatin (276 kDa). The specifications of samples used for SEM imaging are listed in Table S 2.

Table S 2. Specifications of samples subjected to SEM analysis to observe the morphological changes of gelatin with low and high molecular weight upon the addition of acetone.

Acetone volume fractions applied for LMw gelatin	Growth phase	Acetone volume fractions applied for HMw gelatin	Growth phase
67.5	I	63.9	I
68.3	II	69.9	II
72	II	71.9	II
73.7	II	74.4	III
75.2	III		

1.5. Cryo-transmission electron microscopy

To monitor the formation of GNPs in their native hydrated state, cryo-transmission electron microscopy (cryo-TEM) was employed to capture various stages of nanoparticle formation. GNP formation was visualized at three crucial stages of particle formation, i.e. (i) initial acetone-free aqueous gelatin solutions (start of phase I), (ii) gelatin solutions containing 67.5% acetone to validate the formation of primary particles (end of phase I), and (iii) gelatin solutions containing 72% acetone (phase II) to visualize the final GNPs. Gelatin-free acetone-water mixture with an acetone volume fraction of 67.5% was imaged as control. After introducing the required volume fractions of acetone to the gelatin solution at 40 ^{o}C, samples were

vitrified on TEM grids (holey carbon support, Quantifoil) utilizing an automated vitrification robot (FEI Vitrobot™ Mark III, FEI Company). Prior to vitrification, the TEM grids were rendered hydrophilic through glow discharge, using a Cressington 208 carbon coater. A 3 µL drop of each sample was then transferred onto a TEM grid within the environmental chamber (operating at 25 °C with 99% humidity) of the Vitrobot, followed by automatic removal of excess liquid with filter paper. Subsequently, the grid was plunged into liquid ethane, which was maintained at approximately –183 °C. The vitrified sample was then transferred to the cryo-TEM (JEOL TEM 2100) and kept at liquid nitrogen temperatures during imaging. For samples containing acetone, nitrogen slush was utilized instead of liquid ethane for vitrification, as liquid ethane is a good solvent for organic solvents [4]. Using a cryo transfer-holder (Gatan 626), the vitrified sample was then transferred to the cryo-TEM unit which was kept at liquid nitrogen temperature during imaging.

2. Supporting Results

Table S3. Gelatin nanoparticle properties produced by desolvation of two types of gelatins (Mw of 179 (type B) or 276 kDa (type B)) using acetone or ethanol.

Non-solvent	Gelatin Mw (kDa)	Size $_{dry}$ (nm, SEM)	Size $_{hydro}$ (nm, DLS)	Surface charge (mV)
**Acetone**	179	200 ± 84	340 ± 54	-13.9 ± 0.4
**Acetone**	276	129±18	286 ± 9	1.2 ± 0.1
**Ethanol**	179	84±15	254 ± 2	-10.4 ± 0.4
**Ethanol**	276	78±22	201 ± 4	-0.89 ± 0.3

Table S4. Weight fractions of different gelatins and properties of corresponding nanoparticles.

Mw (kDa)	Gelatin type	Subunit (%)	Alpha (%)	Beta (%)	Gamma (%)	Microgel (%)	Size $_{dry}$ (nm, SEM)	Size $_{hydro}$ (nm, DLS)
**179**	B	31	28	16	11	14	200 ± 84	340 ± 54
**229**	A	22	17	23	18	20	161 ± 18	293 ± 15
**244**	A	21	17	23	17	22	152 ± 15	278 ± 21
**276**	A	18	17	22	16	27	129 ± 18	249 ± 9

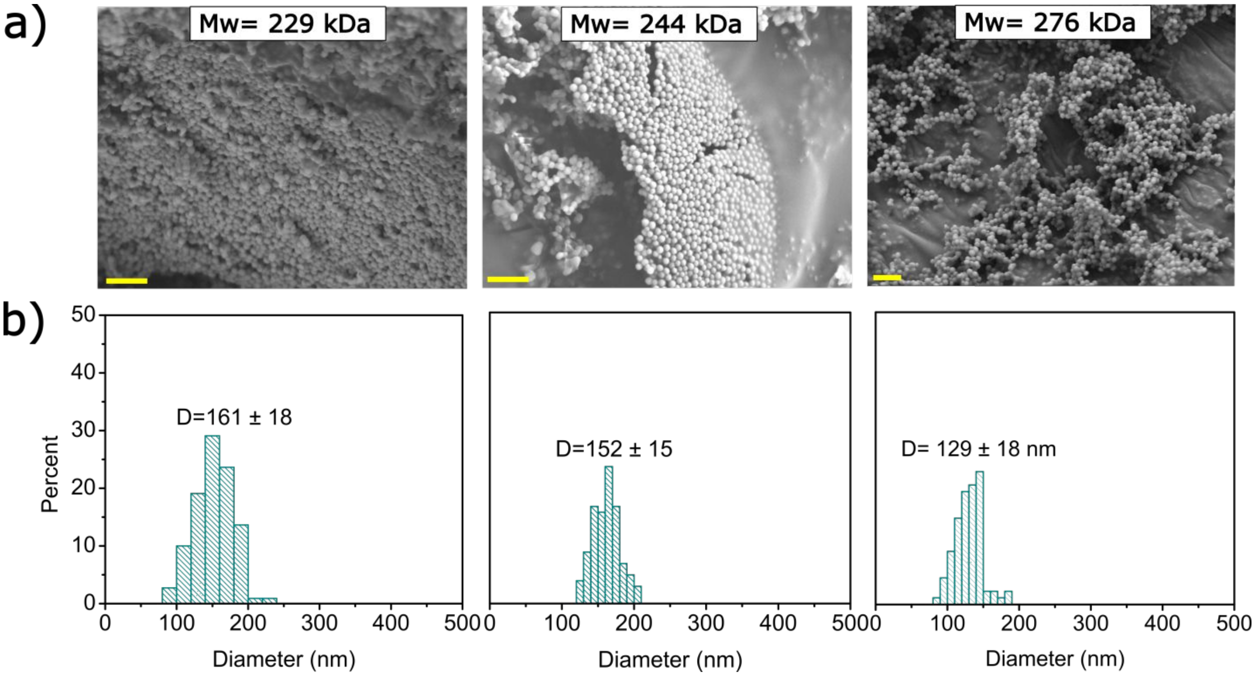

Figure S1. Gelatin nanoparticle characterization. a) scanning electron micrographs of lyophilized GNPs (scale bars correspond to 1µm) produced by gelatins with Mw of 229, 244 and 276 kDa (type A). b) size distribution of lyophilized GNPs obtained from SEM images.

Table S5. Comparison of GNP formation kinetics between LMw-gelatin (type B) and HMw-gelatin (type A) using acetone and ethanol as non-solvent agents. ϕ_{t0} and ϕ_{tm} refer to non-solvent volume fractions corresponding to the onset of the rapid turbidity increase (after phase I) and maximum turbidity (after phase II), respectively.

Non-solvent type	Gelatin type	ϕ_{t0}	ϕ_{tm}	Growth rate
**Acetone**	LMw -gelatin	68.3	73.1	0.67
	HMw - gelatin	64.8	73.1	0.35
**Ethanol**	LMw -gelatin	84.8	87.8	0.13
	HMw - gelatin	77.9	85.2	0.08

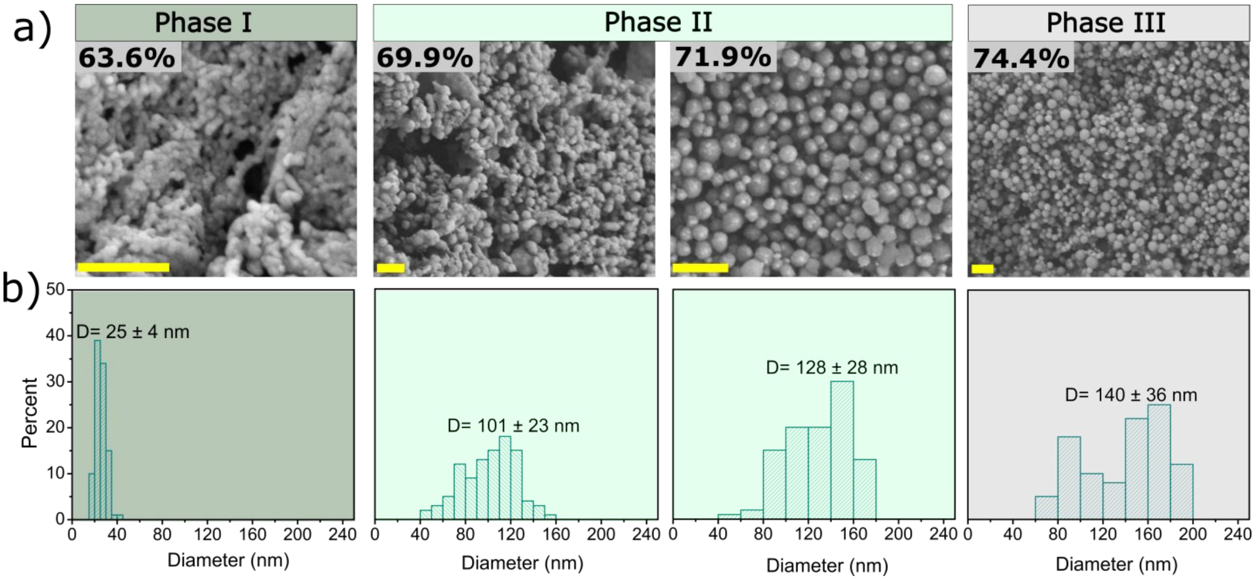

Figure S2. Formation of gelatin nanoparticles from gelatin with a Mw of 276 kDa (type A). a) scanning electron micrographs of freeze-dried gelatin nanoparticles (scale bars represent 400 nm) as a function of acetone content, b) size distribution of freeze-dried gelatin nanoparticles calculated from SEM images.

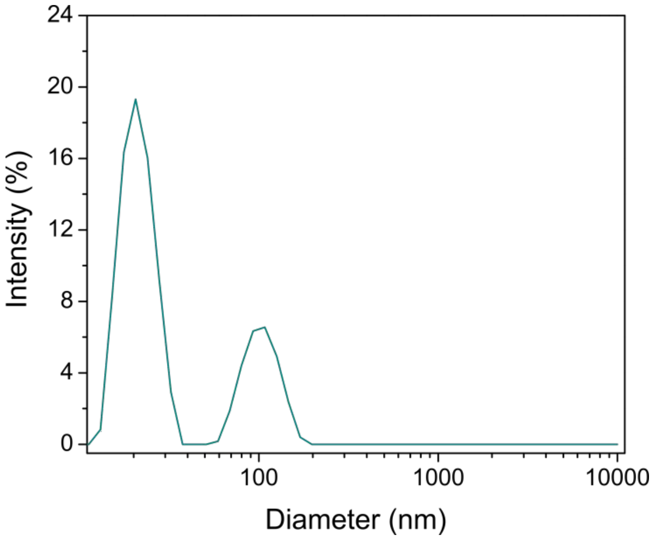

Figure S 3. Size distribution of dissolved gelatin in water (0.001 wt/wt) measured by DLS.

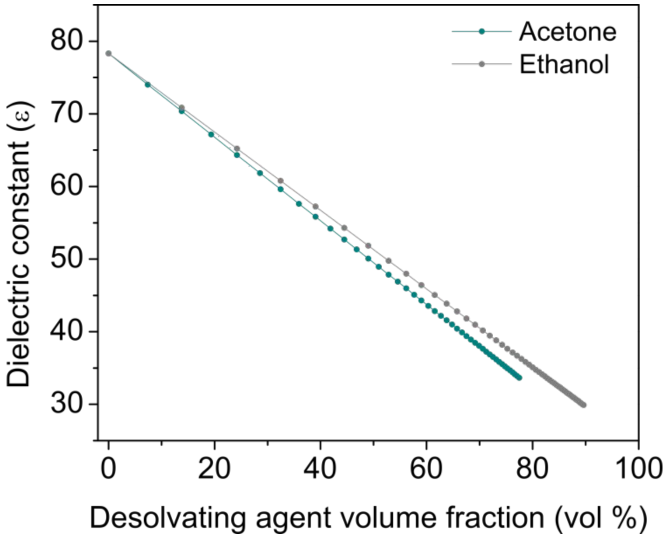

Figure S4. Dielectric constant of mixtures of acetone or ethanol with water as a function of acetone/ethanol volume fraction.

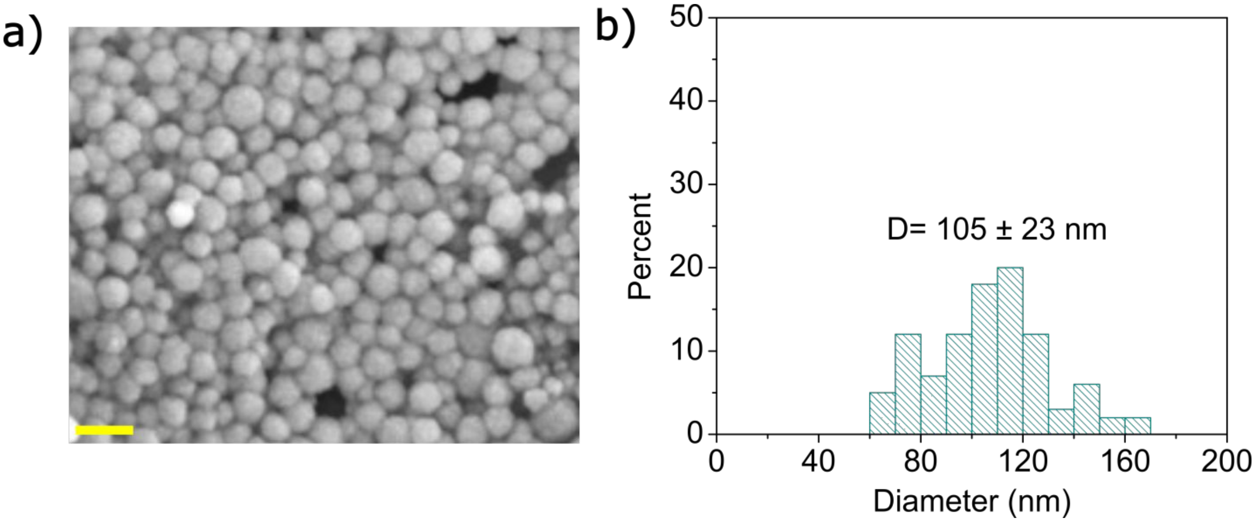

Figure S5. Gelatin nanoparticle characterization. a) scanning electron micrograph (scale bar corresponds to 200 nm) of nanoparticles synthesized from gelatin with a Mw of 179 kDa (type B) and ethanol as desolvating agent. b) size distribution of obtained nanoparticles.

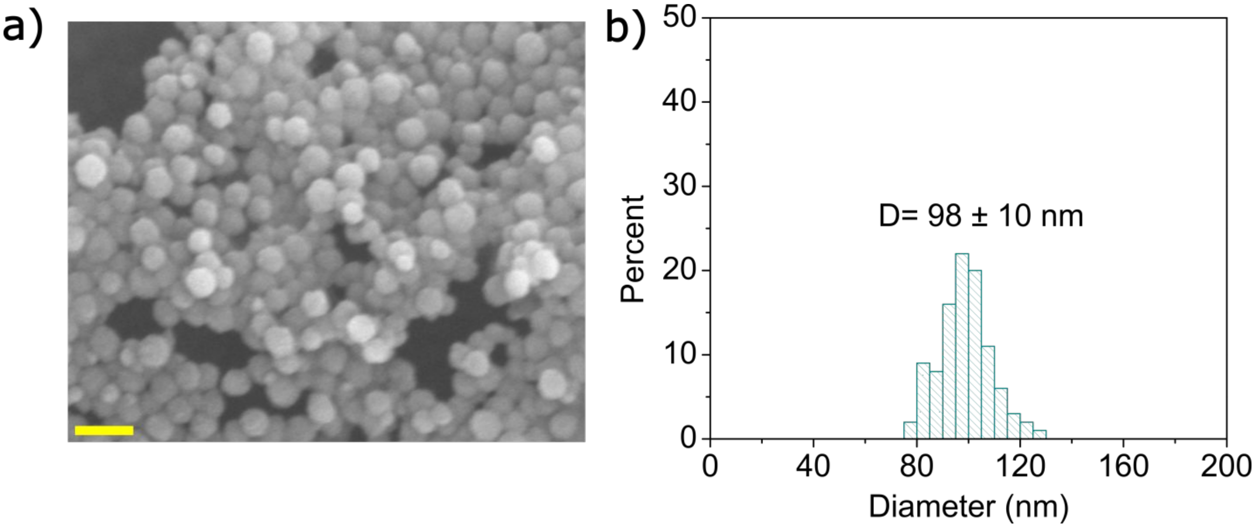

Figure S6. Gelatin nanoparticles characterization. a) scanning electron micrograph (scale bar corresponds to 200 nm) of nanoparticles synthesized from gelatin with a Mw of 276 kDa (type A) and ethanol as desolvating agent. b) size distribution of obtained nanoparticles.

References

1. Geh, K. J.; Hubert, M.; Winter, G., Optimisation of one-step desolvation and scale-up of gelatine nanoparticle production. *J Microencapsul* **2016,** *33* (7), 595-604.

2. Ahsan, S. M.; Rao, C. M., The role of surface charge in the desolvation process of gelatin: implications in nanoparticle synthesis and modulation of drug release. *Int J Nanomedicine* **2017,** *12*, 795-808.

3. Mohammad-Beigi, H.; Shojaosadati, S. A.; Morshedi, D.; Mirzazadeh, N.; Arpanaei, A., The Effects of Organic Solvents on the Physicochemical Properties of Human Serum Albumin Nanoparticles. *Iran J Biotechnol* **2016,** *14* (1), 45-50.

4. Koifman, N.; Talmon, Y., Cryogenic Electron Microscopy Methodologies as Analytical Tools for the Study of Self-Assembled Pharmaceutics. *Pharmaceutics* **2021,** *13* (7).
